# miR-1229-3p promotes epithelial-mesenchymal transition and metastasis of cervical cancer cells by targeting FBXL5

**DOI:** 10.1186/s41065-026-00679-6

**Published:** 2026-04-22

**Authors:** Donglian Lan, Xiaojing Sun, Lei Yao, Bo Cao

**Affiliations:** 1 Department of Gynaecology, Longyan First Hospital, Fujian, 364000 China; 2 Department of Radiotherapy, The Affiliated Hospital of Hebei University, Baoding, 071000 China; 3 Department of Gynaecology, Geriatric Hospital Affiliated to Wuhan University of Science and Technology, No.2, Huangjiahu West Road, Hongshan District, Wuhan, 430065 China; 4 Department of Gynecology Oncology, Hubei Cancer Hospital, Tongji Medical College, Huazhong University of Science and Technology, No.116, Zhuodaoquan South Road, Hongshan District, Wuhan, 430079 China

**Keywords:** Cervical cancer, miR-1229-3p, FBXL5, Epithelial-mesenchymal transition, Cell proliferation

## Abstract

**Objective:**

This study aimed to explore the biological function of miR-1229-3p in the occurrence and development of cervical cancer (CC).

**Methods:**

The expression levels of miR-1229-3p and FBXL5 in CC tissues and cell lines were detected by RT-qPCR. Cell proliferation ability was evaluated by MTT. Cell migration and invasion abilities were detected by Transwell. The expression of EMT-related markers were detected by RT-qPCR. The targeting relationship between miR-1229-3p and FBXL5 was verified by dual luciferase reporter assay.

**Results:**

The expression of miR-1229-3p was significantly upregulated in CC tissues and CC cell lines. High expression of miR-1229-3p was associated with poor differentiation and lymph node metastasis. Overexpression of miR-1229-3p could promote the proliferation, migration, invasion of CC cells, and induce the EMT process. Dual luciferase reporter assays confirmed that FBXL5 was the direct target gene of miR-1229-3p, and their expressions showed a significant negative correlation in CC tissues. Overexpression of FBXL5 could reverse the carcinogenic effects caused by miR-1229-3p.

**Conclusion:**

miR-1229-3p directly inhibits the expression of FBXL5, thereby activating the EMT process, and ultimately promoting the proliferation, migration, and invasion of CC cells.

## Introduction

Cervical cancer (CC) is a malignant tumor that originates from the epithelial cells of the cervix and is one of the most common malignant tumors in the female reproductive system [1, 2]. This disease shows no obvious symptoms in early stage. This leads to some patients being diagnosed only when their condition becomes severe, which greatly increase the difficulty of treatment and the risk of death [3]. CC may lead to the loss of reproductive ability, abnormal pelvic functions, and cause long-term psychological and social burdens [4]. Therefore, conducting in-depth research on the pathogenesis of CC and actively seeking possible targeted therapeutic indicators hold significant clinical importance.

MicroRNAs (miRNAs), as a class of endogenous non-coding small molecule RNAs, regulate gene expression at the post-transcriptional level [5]. During the development of tumors, the dysregulation of miRNA is ubiquitous. This dysregulation either has an inhibitory effect or act as a gene that triggers cancer. Therefore, miRNAs are regarded as important diagnostic markers and potential therapeutic targets [6–8]. Among them, miR-1229-3p is significantly upregulated in the cancer tissues of patients with recurrent CC [9]. It also shows an upward trend in endometrial cancer [10]. In endometriosis, miR-1229-5p significantly promotes the migration of endometrial cells [11]. These studies suggest that miR-1229-3p may be involved in the pathogenesis of CC. However, at present, there is still a lack of systematic research on the specific regulatory mechanism of miR-1229-3p in CC.

FBXL5 is a member of the F-box family. As a key component of the E3 ubiquitin ligase complex, its main function is to precisely regulate cellular homeostasis by recognizing and mediating the ubiquitination degradation of specific proteins [12]. Studies have shown that FBXL5 has inhibitory functions in various cancers [13–15]. In CC, its low expression is closely related to the poor prognosis [16]. FBXL5 can inhibit the EMT by degrading Snail1 and cytoskeletal protein [17]. These findings suggest that FBXL5 may be a potential suppressor factor in tumor. However, the role of FBXL5 in the CC and its interaction network with miR-1229-3p still require further investigation.

Based on the above research background, this study aims to deeply explore the biological function of miR-1229-3p in CC and to reveal whether it targets and regulates the expression of FBXL5.

## Materials and methods

### Participants

This study included 85 patients with CC who were treated at Longyan First Hospital between July 2022 and July 2024. Carcinoma tissue were obtained from patients through surgery, and adjacent normal tissue was also collected as a control sample. The normal tissue was taken from a location at least 3 centimeters away from the edge of the tumor, and it has been confirmed by a pathologist. All samples were placed in cryotubes without ribonuclease after being extracted. The cryotubes were quickly frozen in liquid nitrogen and then transferred to a -80℃ ultra-low temperature refrigerator for long-term storage. The inclusion criteria for cases were as follows: (1) Had not received any anti-tumor treatment; (2) Specimens were obtained through surgery or puncture, and were confirmed by the pathologist to be of the CC type; (3) No radiotherapy or chemotherapy was received before treatment; (4) Complete clinical and pathological data were available; (5) Had no distant organ metastasis.

This study strictly adhered to ethical review norms. The use of all samples was approved by the Ethics Committee of Longyan First Hospital. All patients have signed written informed consent forms.

### Cell culture

Human CC cell lines (HeLa, SiHa, C33A, CaSki) and the normal cervical epithelial cell line (Ect1/E6E7) were obtained from the National Collection of Authenticated Cell Cultures (Shanghai, China). All cells were cultured in DMEM medium (Cat# 11965118, Gibco, USA) and the medium was supplemented with 10% FBS (Cat# 12103 C, Sigma-Aldrich, Germany) and 1% penicillin-streptomycin (Cat#15140122, Gibco, USA). The cells were routinely cultured in a constant temperature incubator at 37 °C with 5% CO₂. The medium was changed and passages were made regularly according to the growth status.

### RT-qPCR

Total RNA was extracted from tissues and cell samples using the TRIzol (Cat# 15596018CN, Invitrogen, USA). The concentration and purity of the RNA were detected using a microplate spectrophotometer. Using the qualified RNA as the template, mRNA and miRNA were reverse-transcribed using the PrimeScript™ RT Reagent Kit (Cat# RR037, TAKARA, Japan) and the miRNA cDNA Synthesis Kit (Cat# CW2141, Cwbiotech, China), respectively. Real-time quantitative PCR was performed on the 7500 fast real-time PCR machine (Applied Biosystems, USA). The quantitative detection of mRNA was performed using TB Green™ Fast qPCR Mix (Cat# RR430, TAKARA, Japan), while the quantitative detection of miRNA was carried out using miRNA qPCR Assay Kit (Cat# CW2142, Cwbiotech, China). The reaction system and cycling conditions were all set in accordance with the instructions. In the experiment, GAPDH was used as the internal reference gene for mRNA, and U6 snRNA was used as the internal reference for miRNA. All data were analyzed by the 2^-ΔΔCt^ method.

### Cell transfection

The miR-1229-3p mimic, mimic controls (mimic NC), miR-1229-3p inhibitor, inhibitor controls (inhibitor NC), pcDNA3.1 plasmid (pc-NC, pc-FBXL5), and small interfering RNA (si-NC, si-FBXL5) used in the experiment were all synthesized by Ribobio Biotechnology (Guangzhou, China). SiHa and C33A cells in the logarithmic growth phase were seeded in 6-well plates at a density of 2 × 10⁵ cells/per well. After the cells adhered to the plate, the overexpression plasmids, siRNAs or miRNA mimic/inhibitor were transfected into cells according to the instructions of the Lipofectamine^®^ 3000 (Cat# L3000008, Invitrogen, USA). Then, the cells were incubated in incubator. After 48 h of transfection, the cells were collected.

### Cell viability assay

SiHa and C33A cells were seeded at a density of 2 × 10³ cells/well in 96-well plate, with each well containing a culture volume of 100 µL. Cell viability was measured at 24, 48, 72 and 96 h after seeding. At each time point, 20 µL of MTT (Cat# CT01, Sigma-Aldrich, Germany) was added, and the cells were incubated 37 °C for 4 h. Then, the supernatant in each well was aspirated, and 150 µL of DMSO was added. The plates were placed on a shaker at room temperature for 10 min. Finally, the OD values of each well were measured using an microplate reader at a wavelength of 490 nm.

### Transwell assays

The invasion experiment was conducted using a Transwell chamber (Cat# CLS3412, Corning, USA). After 48-hours transfection, the cells were digested using trypsin. The cells were centrifuged and the supernatant was discarded. Then, the cells were resuspended in a basic medium without FBS. The cell suspension (1 × 10⁵ cells/well) was added to the upper chamber of the Transwell, and the lower chamber was filled with complete medium containing 10% FBS. After the cells were cultured at 37℃ for 24 h, the cells and matrix gel in the upper chamber were wiped off using a cotton swab. The cells that penetrated to the lower chamber surface were fixed with 4% paraformaldehyde for 15 min, and then stained with 0.1% crystal violet for 20 min. Five random fields were selected under an inverted microscope.The migration experiment was conducted in the same way as the invasion experiment, except that the upper chamber of the Transwell apparatus was not coated with Matrigel matrix gel.

### Dual-luciferase reporter assay

To verify the targeting relationship between miR-1229-3p and FBXL5, bioinformatics analysis was first conducted using the miRDB database. The results showed that the 3’UTR of FBXL5 contains complementary binding sites that match the seed region of miR-1229-3p. At the same time, the TargetScan database was used for cross-validation, and both databases consistently confirmed that FBXL5 is a potential target gene of miR-1229-3p. Based on the binding sites, the luciferase reporter vectors containing the wild-type sequence of FBXL5 (FBXL5-WT) and the corresponding mutated vector (FBXL5-MUT) were constructed. These reporter vectors were co-transfected with miR-1229-3p mimic or the mimic NC into CC cells (SiHa/C33A). After 48 h of transfection, the luciferase activity was determined using the Dual-Luciferase Reporter Gene Assay System (Cat# E1910, Promega, USA).

### Statistical analysis

Statistical analyses were conducted using SPSS and GraphPad Prism software. All data were presented as mean ± SD. Comparisons between two groups were performed using Student’s t-test. For comparisons among multiple groups, one-way ANOVA was used. If the results of the ANOVA indicated significant differences, the Tukey method was employed for post-hoc pairwise comparisons. *P* < 0.05 was considered statistically significant.

## Results

### The expression of miR-1229-3p was upregulated in CC patients and CC cell lines, while the expression of FBXL5 was downregulated

**Fig. 1:**
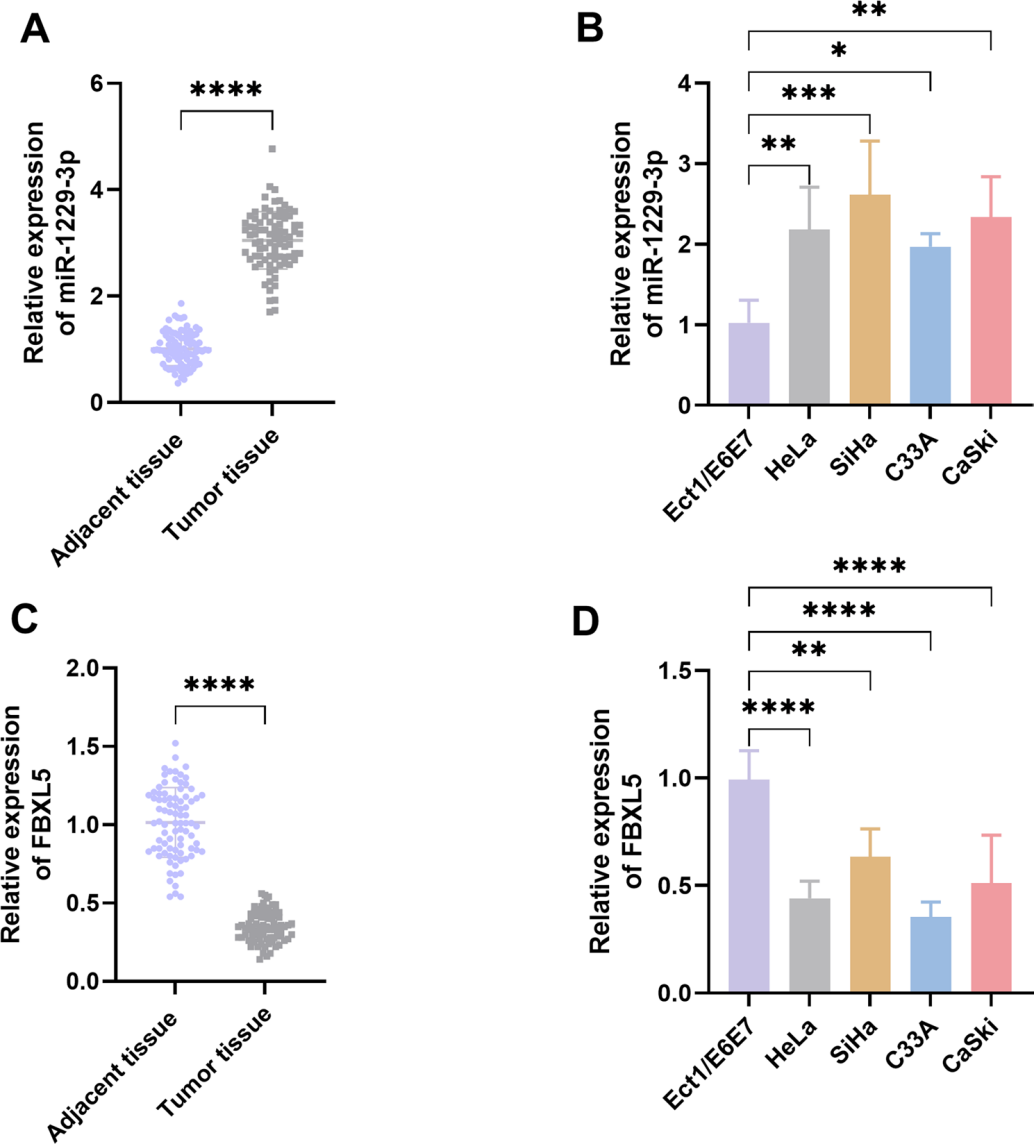
The expression of miR-1229-3p was upregulated in cervical cancer (CC) tissues and CC cell lines, while the expression of FBXL5 was downregulated. **A** The expression of miR-1229-3p in CC tissues. **B** The expression of miR-1229-3p in the CC cell lines. **C** The expression of FBXL5 in the CC tissues. **D** The expression of FBXL5 in the CC cell lines. *n* = 5. * *P* < 0.05, ** *P* < 0.01, *** *P* < 0.001, **** *P* < 0.0001

To investigate the roles of miR-1229-3p and FBXL5 in the progression of CC, we first examined their expression levels in CC tissues and cells. The results showed that the expression level of miR-1229-3p in CC tissues was significantly higher than that in the paired adjacent normal tissues (*P* < 0.0001, Fig. 1A), while the expression of FBXL5 was significantly lower than that in the adjacent tissues (*P* < 0.0001, Fig. 1C). In contrast to the normal cervical epithelial cell line (Ect1/E6E7), the CC cell lines (HeLa, SiHa, C33A, CaSki) exhibited significantly elevated miR-1229-3p levels and reduced FBXL5 expression (*P* < 0.05, Fig. 1B and D). These results indicate that miR-1229-3p and FBXL5 exhibit opposite expression patterns in CC and may respectively play different regulatory roles.

### The relationship between the expression level of miR-1229-3p and clinical parameters

To explore the clinical significance of miR-1229-3p, we analyzed the correlation between its expression level and the pathological characteristics of CC patients. As shown in Table 1, based on the average expression value of miR-1229-3p, the patients were divided into a high-expression group (*n* = 45) and a low-expression (*n* = 40) group. The results of the chi-square test analysis indicated that the high expression of miR-1229-3p was significantly associated with a lower degree of differentiation (*P* = 0.007), a larger tumor size (*P* = 0.038), and lymph node metastasis (*P* = 0.013). However, its expression level was not significantly correlated with the age, histological type, or HPV16/18 infection status. This result suggests that the high expression of miR-1229-3p may promote the malignant progression of the tumor.

**Table 1:** Association between miR-1229-3p levels and clinical characteristics of patients with cervical cancer

Clinical features	*N* = 85	miR-1229-3p expression			*P* value
		Low (*n* = 40)	High (*n* = 45)		
Age (year)					0.759
≤ 45	44	20		24	
> 45	41	20		21	
Differentiation					0.007
Poor	47	16		31	
Well or moderate	38	24		14	
Tumor size (cm)					0.038
<4	43	25		18	
≥ 4	42	15		27	
Histology					0.414
Squamous	63	28		35	
Adenocarcinoma	22	12		10	
HPV16/18					0.443
Negative	42	18		24	
Positive	43	22		21	
Lymph node metastasis					0.013
Negative	41	25		16	
Positive	44	15		29	

### miR-1229-3p promotes the proliferation, migration and invasion of CC cells

**Fig. 2:**
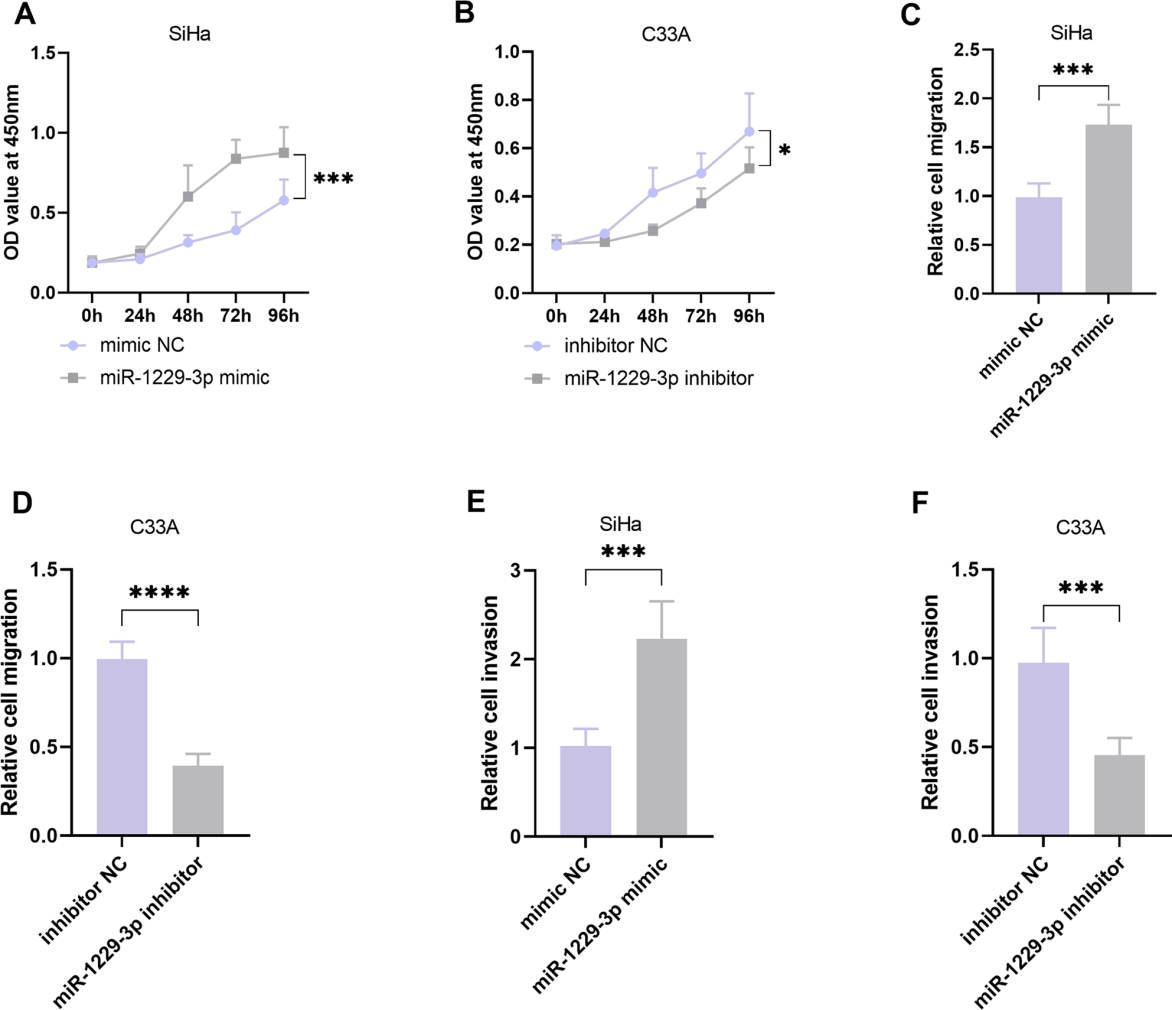
miR-1229-3p promotes the proliferation, migration and invasion of cervical cancer (CC) cells. **A**-**B**. The effect of miR-1229-3p on the proliferation of SiHa and C33A cells. **C**-**D**. The effect of miR-1229-3p on the migration of SiHa and C33A cells. **E**-**F**. The effect of miR-1229-3p on the invasion of SiHa and C33A cells. *n* = 5. * *P* < 0.05, *** *P* < 0.001, **** *P* < 0.0001

We evaluated the effect of miR-1229-3p on the malignant behavior of CC cells through a series of functional experiments. Given that previous tests indicated that miR-1229-3p was expressed at the highest level in SiHa cells and at the lowest level in C33A cells. We respectively selected these two types of cells for the experiment. The MTT experiment showed that miR-1229-3p mimic promoted the proliferation of SiHa cells, while the inhibitor inhibited the proliferation of C33A cells (*P* < 0.05, Fig. 2A, B). The Transwell experiment further demonstrated that the miR-1229-3p mimic significantly enhanced the migration and invasion ability of SiHa cells (*P* < 0.001, Fig. 2C, E), while its inhibitor weakened the corresponding ability of C33A cells (*P* < 0.001, Fig. 2D, F).

### The regulation of miR-1229-3p on EMT-related genes

**Fig. 3:**
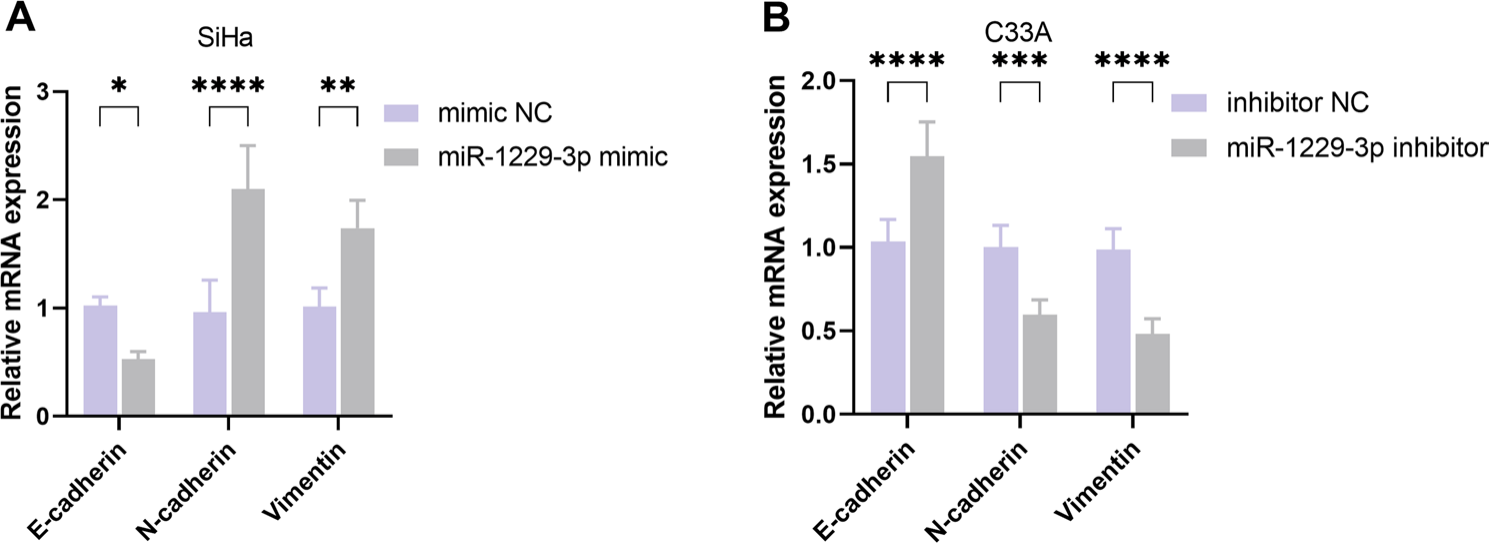
The regulatory effect of miR-1229-3p on the process of epithelial-mesenchymal transition (EMT) in cervical cancer (CC) cells. **A** The mRNA expression levels of E-cadherin, N-cadherin and Vimentin in SiHa cells. **B** The mRNA expression levels of E-cadherin, N-cadherin and Vimentin in C33A cells. *n* = 5. * *P* < 0.05, ** *P* < 0.01, *** *P* < 0.001, **** *P* < 0.0001

To explore the potential mechanism by which miR-1229-3p promotes the migration and invasion of CC cells, we examined its effects on EMT markers. miR-1229-3p mimic in SiHa cells significantly reduced the mRNA level of E-cadherin, while upregulating the expression of N-cadherin and Vimentin (*P* < 0.05, Fig. 3A). In contrast, knockdown of miR-1229-3p in C33A cells led to an increase in E-cadherin and a decrease in N-cadherin and Vimentin (*P* < 0.001, Fig. 3B). These experimental results indicate that miR-1229-3p can induce the EMT process by regulating the expression of EMT-related genes.

### FBXL5 is the downstream target gene of miR-1229-3p

**Fig. 4:**
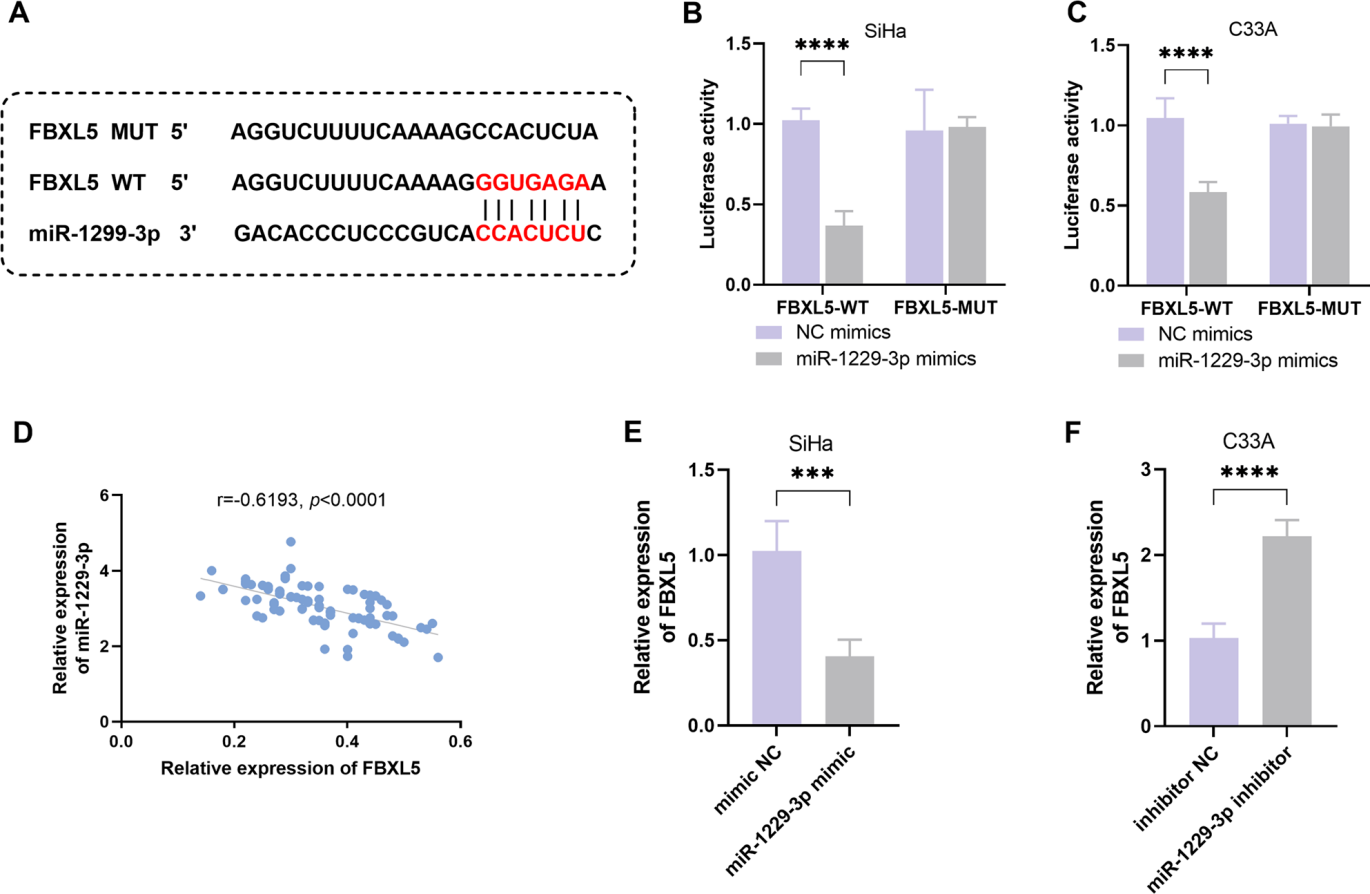
FBXL5 is the downstream target gene of miR-1229-3p. **A**. The binding site between FBXL5 and miR-1229-3p predicted by miRDB. **B**-**C**. The dual-luciferase reporter assay was conducted to verify the interaction between FBXL5 and miR-1229-3p. **D**. The correlation between FBXL5 and miR-1229-3p in the cervical cancer (CC) tissue. **E**-**F**. The regulatory effect of miR-1229-3p on the expression level of FBXL5 in SiHa and C33A cells. *n* = 5. *** *P* < 0.001, **** *P* < 0.0001

Bioinformatics analysis revealed that there are potential binding sites between miR-1229-3p and the 3′ UTR region of the FBXL5 gene (Fig. 4A). To verify this targeting relationship, we conducted a dual-luciferase reporter assay. The results showed that overexpression of miR-1229-3p significantly inhibited the luciferase activity of FBXL5-WT in both SiHa and C33A cells (*P* < 0.0001, Fig. 4B, C). Additionally, in clinical samples, the expression of miR-1229-3p and FBXL5 showed a significant negative correlation (*r* = -0.6193, *P* < 0.0001; Fig. 4D). Overexpression of miR-1229-3p significantly reduced the expression level of FBXL5 in SiHa cells; conversely, inhibiting miR-1229-3p could significantly upregulate the level of FBXL5 in C33A cells (*P* < 0.001, Fig. 4E, F).

### FBXL5 reverses the effect of miR-1229-3p on CC cells

**Fig. 5:**
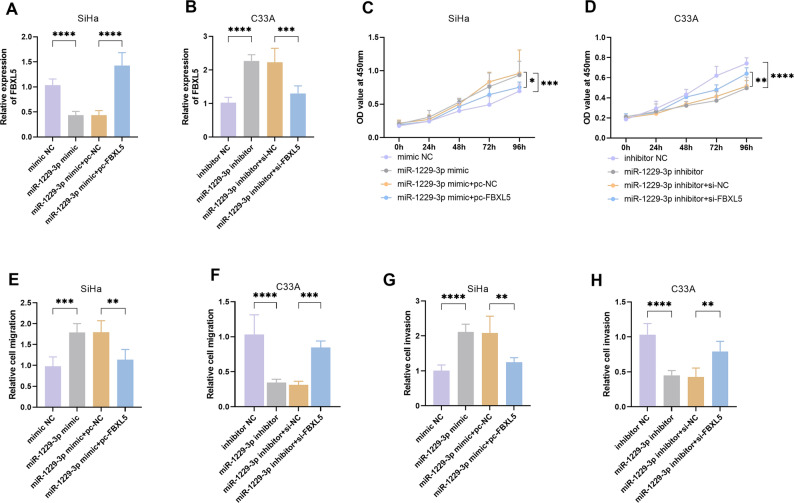
FBXL5 reverses the effect of miR-1229-3p on cervical cancer (CC) cells. **A**-**B**. The expression of FBXL5 mRNA in SiHa and C33A cells. **C**-**D**. The proliferation status of SiHa and C33A cells. **E**-**F**. The migration levels of SiHa and C33A cells. **G**-**H**. The invasion levels of SiHa and C33A cells. *n* = 5. * *P* < 0.05, ** *P* < 0.01, *** *P* < 0.001, **** *P* < 0.0001

To clarify the role of the miR-1229-3p/FBXL5 axis in CC, we conducted a functional rescue experiment. In SiHa cells, miR-1229-3p mimic and FBXL5 overexpression plasmid were co-transfected. The results showed that pc-FBXL5 could significantly restore the downregulation of FBXL5 caused by miR-1229-3p mimic (*P* < 0.0001, Fig. 5A). In C33A cells, when the miR-1229-3p inhibitor was co-transfected with si-FBXL5, the increase in FBXL5 caused by the miR-1229-3p inhibitor was eliminated (*P* < 0.001, Fig. 5B). In SiHa cells, the overexpression of FBXL5 could significantly inhibit the cell proliferation, migration, and invasion caused by miR-1229-3p (*P* < 0.05, Fig. 5C, E, G). In C33A cells, knockdown of FBXL5 significantly reversed the inhibitory effect of miR-1229-3p inhibitor on the above malignant phenotypes (*P* < 0.01, Fig. 5D, F, H).

**Fig. 6:**
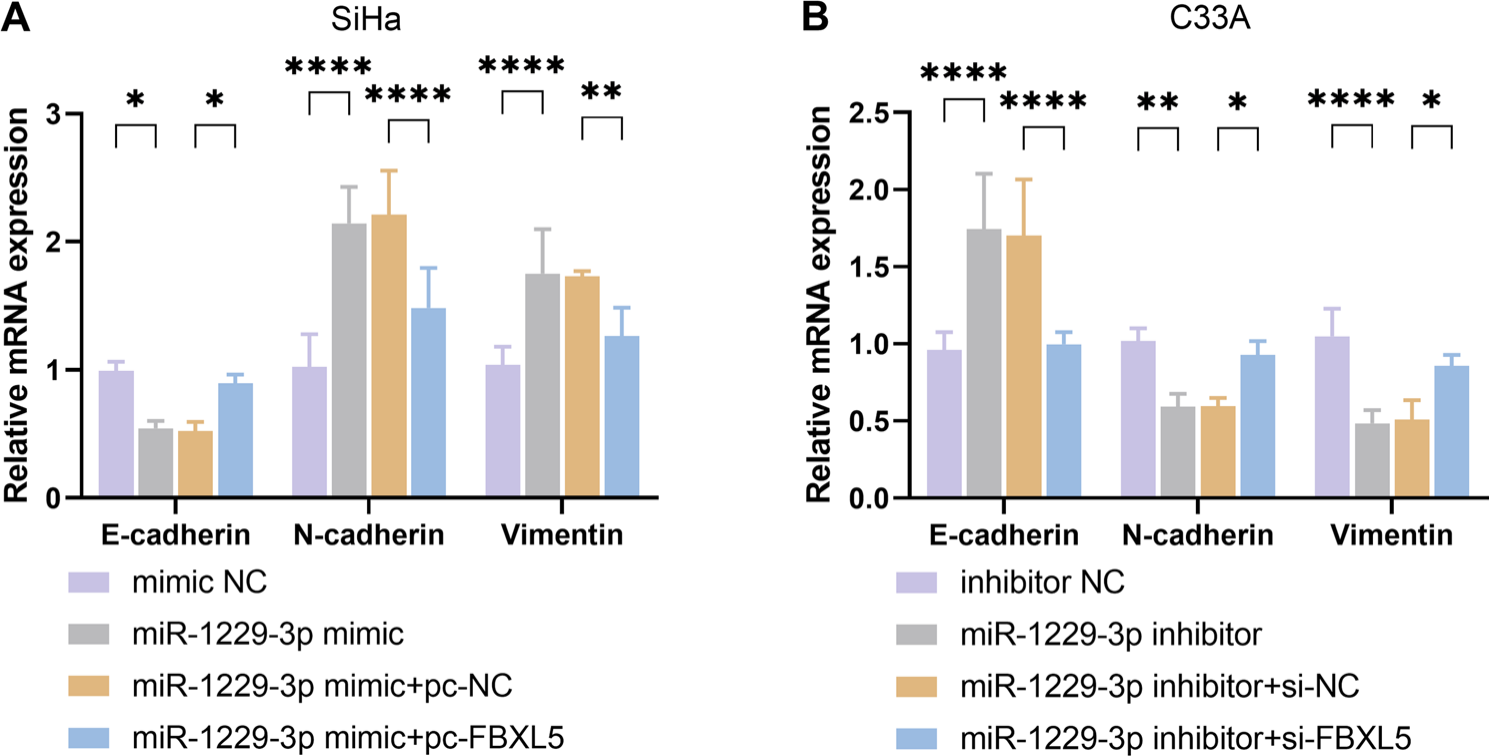
miR-1229-3p promotes the epithelial-mesenchymal transition (EMT) process in cervical cancer (CC) by inhibiting FBXL5. **A** The mRNA expression levels of EMT markers in SiHa cells. **B** The mRNA expression levels of EMT markers in C33A cells. *n* = 5. * *P* < 0.05, ** *P* < 0.01, *** *P* < 0.001, **** *P* < 0.0001

Furthermore, overexpression of FBXL5 was found to reverse the EMT process induced by miR-1229-3p mimic (*P* < 0.05, Fig. 6A). Additionally, knockdown of FBXL5 could counteract the inhibitory effect of miR-1229-3p inhibitor on the process of EMT (*P* < 0.05, Fig. 6B). Our findings indicate that miR-1229-3p drives the malignant progression of CC cells by directly targeting and inhibiting FBXL5.

## Discussion

The pathogenesis of CC is complex, and patients often encounter clinical difficulties such as high risk of recurrence and metastasis. Although various treatment methods such as surgery, radiotherapy, chemotherapy, and targeted therapy have been continuously developed, recurrence and metastasis remain the main challenges in clinical practice [18–20]. Therefore, in-depth research on the pathogenesis of CC and exploration of effective targeted treatment strategies are of great significance for improving prognosis. This study focused on the role of miR-1229-3p and its potential target gene (FBXL5) in CC, and preliminarily revealed the function and mechanism of this regulatory axis in CC.In recent years, with the in-depth research on non-coding RNAs, the value of miRNA in the treatment of CC has received extensive attention [21, 22]. Studies have shown that the expression profile of specific miRNAs can not only serve as a potential biomarker for CC, but also be regarded as an important therapeutic target [23, 24]. For example, miR-21 promotes CC by regulating NTF3 [25]; miR-182 promotes the progression of CC by activating the Wnt/β-catenin pathway [26]. Among these miRNAs, miR-1229-3p’s role in tumors has gradually attracted attention. This study confirmed that miR-1229-3p was significantly overexpressed in CC tissues and cell lines. Moreover, its expression level was closely related to poor tumor differentiation and lymph node metastasis. This result suggests that miR-1229-3p may act as an oncogene in CC. Functional experiments demonstrated that miR-1229-3p could significantly promote the proliferation, migration, and invasion of CC cells. Further mechanism exploration revealed that miR-1229-3p could down-regulate the expression of epithelial marker (E-cadherin) and simultaneously up-regulate the expression of mesenchymal markers (N-cadherin and Vimentin).

To clarify the target of miR-1229-3p, we combined bioinformatics prediction with experimental verification. FBXL5 is its downstream target gene. Research has established FBXL5 as a suppressor in tumor. For instance, in colorectal cancer, FBXL5 maintains iron homeostasis by regulating the IREB2-TFRC axis, thereby inhibiting tumor development [27]; in non-small cell lung cancer, it exerts suppressive function by reducing ROS levels and inhibiting the PI3K/AKT and NF-κB signaling pathways [28]. In CC, it has been reported that the downregulation of FBXL5 is associated with poor prognosis [16]. This study confirmed that overexpression of FBXL5 could significantly reverse the oncogenic effects caused by overexpression of miR-1229-3p. These results systematically indicate that miR-1229-3p mainly drives the malignant progression of CC by targeting and inhibiting the expression of FBXL5.

EMT is a key biological process that enables cancer cells to acquire the ability of migration and invasion [29]. This process is regulated by multiple signaling pathways, such as Wnt/β-catenin and TGF-β [30]. By activating transcription factors such as ZEB and TWIST, it subsequently inhibits the expression of epithelial markers (such as E-cadherin) and promotes the expression of mesenchymal markers (such as N-cadherin, Vimentin) [31, 32]. Existing studies have indicated that FBXL5 can inhibit the EMT process by degrading Snail1 [17]. We infer that the abnormal upregulation of miR-1229-3p directly inhibits the expression of FBXL5, thereby disrupting its negative regulatory effect on EMT. This leads to the abnormal activation of EMT, thereby enhancing the migration and invasion abilities of CC cells. This discovery provides a new perspective for explaining the molecular mechanism of the abnormal activation of EMT in CC. Therefore, we believe that the miR-1229-3p/FBXL5 axis is an important upstream regulatory factor for controlling the EMT process in CC.

This study reveals the significant regulatory role of the miR-1229-3p/FBXL5 axis in CC. The expression level of miR-1229-3p is significantly higher in CC tissues and cells, and it is closely related to unfavorable clinical and pathological features. This indicates that it has the potential to serve as a diagnostic or prognostic biomarker. Furthermore, since miR-1229-3p promotes cell proliferation, migration, invasion and EMT by inhibiting FBXL5, therapeutic strategies targeting the miR-1229-3p/FBXL5 axis are expected to serve as a potential therapeutic entry point. Therefore, in the future, the therapeutic potential of this axis can be verified in animal models, and the possibility of combining it with traditional chemotherapy or immunotherapy can also be explored.

In summary, this study systematically elucidated the oncogenic role of miR-1229-3p in CC. The research results indicate that miR-1229-3p inhibits the expression of FBXL5 through a targeting mechanism, thereby triggering the EMT process and ultimately promoting the proliferation, migration and invasion of CC cells.

### Limitations

This study still has certain limitations. Firstly, the clinical sample size is relatively small and comes from a single center, which may affect the statistical power and the generalizability of the conclusions. Secondly, only cell line models are used for mechanism exploration, while there is a lack of in vivo animal experiment. Moreover, the downstream signaling pathways of the miR-1229-3p/FBXL5 axis has not been deeply elucidated. In the future, a larger sample size should be adopted and combined with in vivo models for analysis to further verify the feasibility of this axis as a therapeutic target.

## Data Availability

The datasets generated during and/or analysed during the current study are available from the corresponding author on reasonable request.
